# Metabolic Profile of Growing Immune- and Surgically Castrated Iberian Pigs Fed Diets of Different Amino Acid Concentration

**DOI:** 10.3390/ani13162650

**Published:** 2023-08-17

**Authors:** Ignacio Fernández-Fígares, Ana Haro, Manuel Lachica, Luis Lara, Isabel Seiquer, Rosa Nieto

**Affiliations:** Department of Nutrition and Sustainable Animal Production, Estación Experimental del Zaidín, Spanish National Research Council, CSIC, Profesor Albareda, s/n, 18008 Granada, Spain; ignacio.fernandez-figares@eez.csic.es (I.F.-F.); ana.haro@eez.csic.es (A.H.); manuel.lachica@eez.csic.es (M.L.); luis.lara@eez.csic.es (L.L.); isabel.seiquer@eez.csic.es (I.S.)

**Keywords:** gonadotrophin releasing hormone, growth, local fatty pig, IGF-1, insulin, metabolism, plasma free amino acids

## Abstract

**Simple Summary:**

Pig immunocastration—vaccination against the gonadotropin-releasing hormone—prevents sexual development and boar taint, an unpleasant odor perceived in pork products. It is a feasible alternative to surgical castration that considerably increases pig well-being. Apart from this benefit, immunocastrated pigs perform better than surgically castrated pigs, which is of interest for pig production in general, and for native pig breeds in particular, as they show slow growth and lean tissue deposition compared to cosmopolitan pig breeds. These performance benefits were demonstrated in immunocastrated Iberian male pigs, a local pig breed from the Iberian Peninsula. The purpose of the study was to investigate the metabolic profile of immunocastrated male and female Iberian pigs compared to surgically castrated male pigs, which is the standard pig type for Iberian pig production. The surgically castrated males showed metabolic features of pigs prone to deposit more lipids, with less lean tissue and they were less sensitive to circulating insulin, whereas immunocastrated males presented increased plasmatic concentrations of some key amino acids and hormones (IGF-1) related to enhanced protein deposition. The immunocastrated female group showed intermediate traits. The results support the higher performance previously observed in immunocastrated male Iberian pigs compared to surgically castrated males and immunocastrated females.

**Abstract:**

The purpose of the current study was to further characterize the performance and nitrogen retention differences previously observed between immunocastrated (IC) and surgically castrated (SC) pure Iberian pigs. Fifty-four pigs were used (three sexes: IC males, IC females and SC males), fed three isoenergetic diets (160, 140 and 120 g CP/kg DM; six pigs/treatment combination) from 40 kg BW until slaughter (105 kg BW). Plasmatic post-absorptive metabolites and hormones, and backfat tissue composition were determined. The IC males showed a trend towards higher plasmatic creatinine (*p* = 0.06) and IGF-1 concentrations than SC males and IC females (*p* < 0.001). SC males showed a higher predisposition to carcass fatness and insulin resistance compared to IC males. Plasmatic isoleucine concentration was higher in IC and SC males compared to IC females (*p* < 0.01), and valine was greater in IC males compared to the rest of the pigs (*p* < 0.001). Total branched-chain amino acids (AA) were greater in IC males than in IC females (*p* < 0.01). Total plasmatic essential AA concentrations tended to be greater in IC males (*p* = 0.09). The present results support the increased performance and nitrogen retention capacity previously observed in IC male Iberian pigs compared to SC males and IC females, which can be attributed to increased anabolic capacity related with lean growth in Iberian IC males.

## 1. Introduction

One of the current issues that pig production faces is the controversy around the physical castration of male piglets to prevent boar taint, an unpleasant odor perceived in pork and pork products. This traditional practice raises increasing welfare concerns [1], and it is banned in several European countries if not performed with pain relief even during the first days of life [2]. Alternatives to surgical castration are immunocastration or the production of entire males [3]. Immunocastration is the vaccination against gonadotropin releasing hormone (GnRH), a process that suppresses the hypothalamic–pituitary–gonadal axis [4,5], preventing sexual development and boar taint in male pigs [6,7]. The production of entire males is not suitable in heavy pigs raised to obtain high-quality products [8], as is the case with Iberian and other native pig breeds reared in extensive or semi-extensive conditions [9]. Female Iberian pigs may also be gonadectomized to prevent unwanted estrus or pregnancies during outdoors rearing [10,11].

The vaccination protocol of GnRH consists of two subcutaneous doses at least 4 weeks apart (the last one usually 4 to 6 weeks prior to slaughter). GnRH antibody production is boosted after the second dose, causing the suppression of testicular function [6,7,12] and the ovarian cycle [13,14]. Before receiving the second dose of the vaccine, immunocastrated (IC) male pigs could be considered as boars, and therefore, better performance compared to surgically castrated (SC) males is expected [7,15]. Increasing N retention and suppressing physical castration are key issues to improve the environmental sustainability of pig production [16]. 

Apart from the noticeable benefits in animal welfare, higher growth rates could be advantageous for autochthonous breeds—such as the Iberian pig—characterized by reduced lean growth capacity [17]. In a recent study comparing Iberian IC males and females and SC males pigs, we showed that IC males grew at a faster rate, and deposited more protein at a more efficient rate before the second vaccination against GnRH than SC males [18]. Iberian IC females presented similar growth to SC males and intermediate protein deposition efficiency [18]. Although differences in protein deposition and the efficiency of nitrogen retention between sex groups disappeared after the second vaccination, daily gain and feed efficiency were still higher in IC vs. SC males considering the whole fattening period. Furthermore, some lean components of the carcass (loin, sirloin and butt lean) and the carcass length were greater in IC vs. SC males, whereas kidney fat and some backfat thickness measurements were decreased, and backfat androstenone and skatole levels showed similar concentrations in IC and SC males at slaughter—5 weeks after the second vaccination [18].

However, few studies have addressed the dynamics of nitrogen metabolism in IC pigs [19,20,21,22], an essential step for establishing nutritional requirements and adopting feeding programs suited to IC pigs [21,22,23]. Moreover, the mechanisms involved in the metabolic changes underlying differences in nutrient utilization and protein deposition dynamics between IC, SC and entire male pigs have been little investigated. Le Floc’h et al. [24] evaluated the nutrient plasma profile after a meal in catheterized SC, IC and entire male pigs, aiming at explaining some of the metabolic differences among sex groups. They suggested that the decrease in sexual hormones impacted energy metabolism more rapidly than protein metabolism [24].

In the case of autochthonous pigs, we are not aware of studies assessing nitrogen metabolism and protein deposition in IC pigs apart from the aforementioned in Iberian pigs [18]. The purpose of the current study was to further characterize the performance and nitrogen metabolism differences observed between IC and SC pure Iberian pigs. We hypothesized that Iberian IC pigs differ in metabolic traits from SC male pigs five weeks after the second vaccination against GnRH. Both IC males and females were compared with SC males, the reference group as the standard for Iberian pig production. In addition, the effects of feeding increasing levels of dietary amino acids (AA) were tested to check differences in metabolic traits between IC pigs and SC males regarding AA intake. Pigs were reared in intensive conditions until approximately 105 kg BW, resembling production conditions prior to outdoor finishing (*montanera*), wherein feeding is based on free access to acorns and pasture [9]. The main plasma metabolites and hormones and backfat fatty acid (FA) profiles were tested to unravel metabolic changes and the different potential for lean growth between the sex groups. These aspects could be of relevance to the Iberian and other native pig breeds susceptible to immunocastration as a strategy for increasing animal welfare and avoiding boar taint; at the same time, these pigs will benefit from a transient period of better performance and capacity for lean growth in contrast to surgical castration. 

## 2. Materials and Methods

### 2.1. Animal, Diets and Experimental Design

The experimental design has been fully described previously (Palma-Granados et al., 2021 [18]). In brief, 54 pure Iberian males and gilts (Sánchez Romero Carvajal Jabugo, Puerto de Santa María, Cádiz, Spain) were allocated to a 3 × 3 factorial arrangement otreatments with 3 sex groups (IC males, IC females and SC males), and 3 isoenergetic diets (150, 130 and 110 g crude protein (CP)/kg dry matter (DM); 14 MJ metabolisable energy (ME)/kg DM), with 6 pigs per combination of treatments. Two males and one female piglet per litter were selected at birth. Surgical castration of males was performed within the first week of life. At approximately 40 kg BW and 18 weeks of age entire males and females were vaccinated against GnRH with Improvac^®^ (Zoetis, Madrid, Spain) and all pigs were allocated to one of the three experimental diets blocked by BW. The diets were based on barley, maize and soybean meal, added with synthetic AA to achieve an adequate AA profile [25] (Supplementary Table S1). Three levels of protein were used. The intermediate protein level was optimal for Iberian SC males according to previous studies (130 g CP/kg DM, of “ideal” AA ratios; 6.86 g digestible protein/MJ ME, as described in Nieto et al., 2012 [17]), one CP level above (150 g CP/kg DM) and one below (110 g CP/kg DM) were also included. These CP levels were maintained thorough the whole study to facilitate the experiment handling. Pigs were fed twice daily (8:00 and 15:00) at 0.9 × ad libitum on a BW basis determined weekly [17] and had free access to water. Individual feed intake was recorded daily for all pigs. Seven weeks after the first vaccination the pigs received a second dose of the vaccine (70–80 kg BW), and at approximately 105 kg BW (5 weeks after the second vaccination) the pigs were slaughtered by exsanguination after electronarcosis following an overnight fast. Blood samples were taken in EDTA-containing tubes, placed immediately in an ice bath and centrifuged at 1400× *g* at 4 °C for 20 min. Plasma was aliquoted and stored at −80 °C until analyses. Subcutaneous backfat samples (5–10 g) were taken from the right half-carcass at the first lumbar vertebra level and stored at −80 °C until analyses. The experiment was performed in two replicates with 27 pigs each. 

### 2.2. Sample Analyses 

#### 2.2.1. Plasma Metabolites and Hormones

Plasma glucose, triglycerides, ammonia, cholesterol, creatinine and urea were colorimetrically determined in duplicates using a COBAS INTEGRA 400 analyzer (Roche Diagnostics GmbH, Mannheim, Germany). Plasma insulin (Porcine Insulin RIA, catalog number PI-12K, EMD Millipore Corporation, Madrid, Spain), IGF-1 (IGF-1 RIA catalog number IGF-R20, Mediagnost, Reutlingen, Germany), leptin (Multi-Species Leptin RIA, catalog number XL-85K, EMD Millipore Corporation, Madrid, Spain), and total ghrelin (Total Ghrelin, catalog number GHRT-89HK, EMD Millipore Corporation, Madrid, Spain) were determined in duplicates using commercial RIA kits following manufacturer instructions as explained previously [26]. Radioactivity in samples was measured using a γ counter (Behring 1612; Nuclear Enterprises Ltd., Edinburgh, Scotland, UK). The intra-assay CV was 9.3% for insulin, 7.2% for leptin, 9.6% for IGF-1, and 8.8% for total ghrelin. Sensitivity for insulin, leptin, IGF-1, and total ghrelin was 1.611 microunits/mL, 0.801 ng/mL, 0.02 ng/L and 93 pg/mL, respectively.

Insulin sensitivity was estimated according to human medicine indexes that have been additionally used in the animal science context, using the homeostasis model assessment (HOMA [27]) to obtain insulin resistance (HOMA-IR) and β-cell function (HOMA-%B) under fasting conditions:

HOMA-IR = fasting plasma insulin (μU/mL) × fasting plasma glucose (mM)/22.5

HOMA-%B = (20 × fasting plasma insulin (μU/mL))/(fasting plasma glucose (mM) − 3.5)

The quantitative insulin sensitivity check index (QUICKI [28]) was estimated following the formula:

QUICKI = 1/[Log(I0) + Log(G0)]

where I0 is fasting insulin (μU/mL) and G0 is fasting glucose (mg/dL).

#### 2.2.2. Free Amino Acid (AA) Concentrations in Plasma

Plasma free AA concentrations (μmol/L) were analyzed by HPLC according to the Waters Pico Tag method for free AA [29] with pre-column derivatization with phenylisothiocyanate, using a Waters 2695 separation module (Waters Cromatografía, SA, Madrid, Spain) as described previously [30]. Before derivatization, 500 μL of plasma was deproteinized by diluting 1:1 with 20% trichloroacetic acid and 2 mM norleucine as an internal standard, and centrifuged at 11,000× *g* and 4 °C for 15 min. A Millennium 32 chromatography manager system was used for gradient control and data processing.

#### 2.2.3. Total Fat and Fatty Acid (FA) Analyses in Subcutaneous Backfat Samples

Crude fat content and FA determination was performed as described in Palma-Granados et al. [26]. Briefly, crude fat was determined by extraction of 2 g of tissue with chloroform/methanol (2:1, *v*/*v*) according to Folch et al. [31]. Fatty acids were extracted as described by Folch et al. [31] with small modifications. The methylation of FA was performed following the procedure of Kramer and Zhou [32], slightly modified: two methylation procedures were used in each sample, NaOH/methanol at 50 °C for 15 min, followed by HCl/methanol at 50 °C for 1 h. Tricosanoic acid (C23:0; Larodan Fine Chemicals, Malmö, Sweden) was used as the internal standard. Fatty acids methyl esters were identified by gas chromatography using a gas cromatograph equipped with a flame ionization detector (Focus GC, Thermo Scientific, Milan, Italy) and a capillary column (Teknokroma, phase: TR-CN100, 100 m × 0.25 mm i.d. and 0.20 µm film thickness). The gradient temperature program used was: 70 °C, 4 min, ramp of 8 °C/min to 110 °C, ramp of 5 °C/min to 170 °C, 10 min at 170 °C, ramp of 4 °C/min to 240 °C and 240 °C for 14.5 min (total time 63 min). The injector and detector were maintained at 255 °C. The carrier gas (helium) flow rate was 1.2 mL/min with a split ratio of 50, and 1 µL was injected. Areas of peaks were quantified based on the reference standard mixture, with correction for recovery of the internal standard. The FA profile was expressed as a percentage of identified FA relative to total FA.

### 2.3. Statistical Treatment

The statistical treatment of data was assessed by analysis of variance using the GLM SAS procedure [33]. The effects of sex group, dietary protein, trial replicate, and their interactions were included in the statistical model. The trial replicate effect was found not to be significant, and it was therefore removed from the model and the data were reanalyzed. The individual pig was the experimental unit for all measurements. The level of significance was set to 0.05 and a tendency of significance was considered for *p*-values between 0.05 and 0.10. There were no significant sex group × dietary protein interactions for any of the variables analyzed.

## 3. Results

Growth performance and nitrogen retention parameters have been published elsewhere [18]. In brief, before the second vaccination, Iberian IC males showed higher growth rate (g/day), feed efficiency, nitrogen retention (g/day) and efficiency of nitrogen retention than the other groups (*p* < 0.001). Compared to SC males, nitrogen retention and the efficiency of nitrogen retention were 40% greater in IC males (*p* < 0.001). After the second vaccination, no differences in growth performance between sex groups were found (*p* > 0.05), although growth rate and feed efficiency were higher (*p* < 0.001) in IC males than in the other groups considering the whole fattening period (from the first vaccination to slaughter); differences among sex groups in nitrogen retention disappeared (*p* > 0.05). No significant effects of dietary protein levels were detected on performance, although nitrogen retention was greater in pigs fed the higher protein content diet (153 g CP/kg DM; *p* < 0.05). 

### 3.1. Plasma Metabolites and Hormones

The results concerning the concentration of metabolites and hormones in plasma are shown in Table 1. Plasma glucose tended to be lower in IC vs. SC male pigs, with IC females showing intermediate values (*p* = 0.077). Total cholesterol, HDL-cholesterol and LDL-cholesterol were—or tended to be—lower in IC males compared to SC males, with IC females in an intermediate position (*p* < 0.05 for LDL-cholesterol; *p* = 0.063 and 0.066 for total and HDL-cholesterol, respectively). Plasma creatinine showed a strong trend to be greater in IC vs. SC males (IC males > IC females > SC males; *p* = 0.055). No significant differences among sex groups were observed for plasma triglycerides, ammonia and urea concentrations (*p* > 0.05). Dietary protein had little influence on the metabolites analyzed, except for decreased plasma glucose in pigs fed the intermediate protein diet (137 g CP/kg DM; 17% lower glucose on average; *p* < 0.05), and a progressive increase in plasma urea as the dietary protein increased (*p* < 0.001). 

Regarding plasma hormone concentrations, IC males showed lower insulin (53%) than SC males, and IC females had intermediate values (*p* < 0.05). IC males had higher IGF-1 than SC males and IC females (26–45% higher; *p* < 0.001) and lower leptin concentrations (35–43% lower; *p* < 0.01). A trend towards higher values in total ghrelin in IC males compared to the other two groups was observed (*p* = 0.057). SC males showed a higher index of insulin resistance (189% HOMA-IR; *p* < 0.05) and lower for insulin sensitivity (9% lower QUICKI; *p* < 0.01) compared to IC males, with IC females in an intermediate position. No significant differences between groups were observed for estimations of β-cell function (HOMA-%B; *p* > 0.05). No effects of dietary protein on hormone levels were observed apart from the greater IGF-1 in pigs fed the higher protein diet (19%; *p* < 0.05).

### 3.2. Free AA Concentrations in Plasma

The effects of immunocastration and dietary protein intake on concentrations of free AA in plasma are shown in Table 2. In general terms, the most abundant essential AAs were the branched-chain AAs (BCAA; valine, isoleucine and leucine) and lysine, whereas glycine, glutamic acid, alanine, glutamine and proline achieved the highest concentrations among non-essential AAs. The effects of immunocastration on plasma AA concentration were moderate. Histidine concentration was greater in IC males than in SC males (32%; *p* < 0.01), with IC females in an intermediate position. Isoleucine was higher in both type of males compared to females (14%; *p* < 0.01), and valine was greater in IC males compared to SC males and IC females (17%; *p* < 0.001). Total BCAA levels were greater in IC males than in IC females (16%; *p* < 0.01), and SC males had intermediate values. For total essential AA, there was a trend for higher AA concentrations in IC males > SC males > IC females (*p* = 0.096). Regarding non-essential AA concentrations, alanine was higher in IC females compared with both groups of males (*p* < 0.05). The IC males had—or tended to have—lower concentrations of glycine (*p* = 0.09), proline (*p* < 0.05), phosphoserine (*p* < 0.001) and taurine (*p* < 0.001) compared to SC males and IC females. Total non-essential AA (*p* < 0.01) and total non-proteinogenic AA (*p* < 0.05) were lower in IC males compared to females, and SC males had intermediate values. No significant differences between sex groups were detected for proteinogenic and total plasma free AA (*p* > 0.05). Dietary protein level had little influence on plasma AA profile; lysine concentration tended to be lower in pigs fed the highest protein diet (*p* = 0.051), and the same was observed for tyrosine (*p* = 0.055) and total non-essential AA (*p* = 0.072). Glycine concentration was highest in pigs fed the diet with an intermediate protein level (137 g CP/kg DM; *p* < 0.05).

### 3.3. Total Fat and Fatty Acid (FA) Analyses in Subcutaneous Backfat Samples

The results corresponding to the lipid contents and FA composition of backfat samples are depicted in Table 3. Total lipids in backfat were reduced in IC males compared to SC males and IC females (1.8%; *p* < 0.001). C14:0 was lower in IC males vs. SC males (*p* < 0.05), and C18:1 n-7 was reduced in IC females with respect to both groups of males (*p* < 0.01). The concentration of C18:2 n-6 was higher (9%) in IC males and females vs. SC males (*p* < 0.01); C20:2 n-6 and C20:4 n-6 concentrations were higher in IC females than in SC males, and IC males had intermediate values.

The concentrations of C20:3 n-6 and C22:0 were higher in IC females than in both groups of males (*p* < 0.05 and <0.01, respectively); IC males showed lower values of C22:4 n-6 than SC males and IC females (*p* < 0.01). There were no differences among sex groups for total saturated and total monounsaturated FA, although total polyunsaturated and total n-6 FA were greater in IC males and females compared to SC males (7.8 and 9.4% higher, respectively; *p* < 0.01). The n-6/n-3 ratio was higher in IC males compared to SC males (*p* < 0.01). Regarding protein intake levels, C18:3 n-3 was greater in pigs fed the highest protein content vs. pigs fed the lowest-protein diet (*p* < 0.05). C22:5 n-3 was greater in pigs fed the highest-protein diet with respect to the rest of pigs, whereas C20:0 was decreased in pigs fed the highest-protein diet. The n-6:n-3 ratio was the highest in pigs fed the lowest-protein diet (18%; *p* < 0.05).

## 4. Discussion

The main objective of finding alternative options to the physical castration of pigs is to attain high standards of animal welfare, a current challenge for pig production systems. Nevertheless, immunocastration can provide additional productive benefits apart from pig wellbeing, such as a pronounced anabolic state induced by androgens and estrogens [34] maintained during a longer period of time up to the second vaccination. 

The need for an effective method to eliminate boar taint and the possibility of increased performance and capacity for lean growth make immunocastration an attractive approach for autochthonous pig breeds—such as the Iberian—with reduced capacity for protein deposition and lower nutrient utilization efficiency compared to cosmopolitan breeds [17,35]. A limitation of our study is that the SC female control group could not be included in the experimental design. However, other authors showed similar daily gain, feed efficiency and feed intake between SC Iberian males and females [36]. As surgically castrated males are considered the standard for Iberian pig production, IC males and females were compared with SC males as the reference group. Metabolic differences of the three sex groups were investigated five weeks after the second vaccination at approximately 105 kg BW, corresponding to the starting point of outdoor finishing when pigs consume acorns and pasture ad libitum, depending on the availability of natural resources [37]. Plasma metabolites, hormones and free AA concentrations, along with backfat tissue FA composition, were analyzed to further characterize the superior performance and nitrogen retention of IC vs. SC pure Iberian pigs previously reported [18]. We are not aware of similar studies concerning a native pig breed.

Postabsorptive plasma parameters showed a higher aptitude for fat accumulation in SC males compared to IC males, with higher plasma cholesterol and lower creatinine, similarly to previous observations comparing the plasma metabolites of fatty and lean pig types [26,38]. The composition of the backfat tissue corroborated this observation. Lower total lipid content and higher concentrations of polyunsaturated FA—linoleic acid and its derivatives in particular—in the backfat of IC males indicate reduced de novo lipid synthesis [39] compared to SC males, a common observation when pigs of leaner types are compared with fatty ones [40,41]. Similar differences in backfat fatty acid composition have been described for IC vs. SC males and for both vs. entire males, consistent with differences in carcass fatness [42,43]. The plasma metabolites and backfat tissue FA composition values of IC females in the present study were between IC and SC males. 

A trend towards higher plasma glucose, the main carbon source for de novo lipogenesis, was observed in SC males compared to IC males. Greater plasma insulin concentration and HOMA-IR and reduced QUICKI in the SC group compared with IC males (+100%, +290% and −9%, respectively) suggests insulin resistance. Again, intermediate values were found for IC females. The higher plasma cholesterol and insulin resistance of SC males vs. IC males resemble the findings of Torres-Rovira et al. [44], who compared the metabolic status in Iberian pigs freely eating a diet enriched in saturated fat vs. control Iberian pigs receiving a conventional diet. Christoffersen et al. [45] observed in an experiment with male Göttingen minipigs that there was no significant change in insulin sensitivity 10–18 days after surgical castration in comparison with entire males, although insulin sensitivity decreased 10–11 weeks post-castration. In contrast, Le Floc´h et al. [24]—in a study aiming at explaining metabolic differences among sex groups—did not find differences in postprandial plasma insulin profile in entire, SC, and IC Piétrain × (Large-White × Landrace) male pigs two weeks after the second vaccination. 

The metabolic profiles of SC male pigs found in the present study, and their increased carcass fatness vs. IC males previously reported [18], are consistent with the higher levels of plasmatic leptin detected in SC males. It is well-known that there is a positive relationship between plasmatic leptin and the amount of body fat [46]. However, IC Iberian females also showed higher leptin levels than IC males, although carcass fatness measurements did not differ [18]. Batorek et al. [47] also found higher leptin in the plasma of SC vs. IC males fed either restrictedly or ad libitum, and in both SC and IC males, plasmatic leptin was higher than in entire males, although differences between groups were much smaller than in the present case. These authors related the small decrease in serum leptin of IC vs. SC to the higher intake capacity observed in IC males after the second vaccination [47], as leptin levels are known to reduce voluntary feed intake [48]. Beside this, in the present work, plasmatic total ghrelin levels tended to be higher in IC males vs. SC males and IC females. This peptide, which is produced mainly in the stomach, could act as an appetite-regulatory signal, among other functions [49]. Despite differences in plasmatic leptin and ghrelin between IC males and the other two groups of the present work, differences in feed intake were negligible, and all groups maintained high intake levels (nearly 4 times the maintenance metabolizable energy intake [18]). The mechanisms of intake regulation in Iberian pigs may well differ from those of cosmopolitan pigs, as high intake capacity [50] and leptin resistance related to a polymorphism of the leptin receptor gene have been described for this local breed [51].

In the present work, IC males had greater plasmatic levels of IGF-1 than SC males (45%) and IC females (26%). Other authors also showed that after the second vaccination IGF-1 was higher in IC v. SC males [12,24,47], although it was lower compared to entire males. On the other hand, Metz and Claus [52] did not detect differences in IGF-1 levels between IC and SC males, probably due to differences in experimental conditions, including vaccination protocol (three injections starting at week 10 of age). No information on plasmatic levels of IGF-1 in IC females has been found. 

Plasmatic anabolic steroids, including androgens (as testosterone) and estrogens—present also in high amounts in boars—decrease rapidly after the second vaccination [24,34]. Androgens and estrogens both contribute to the anabolic potential, with androgens stimulating protein synthesis and decreasing protein degradation, whereas the anabolic effect of estrogens is related to the stimulation of plasmatic levels of IGF-1 enhancing protein synthesis rates [34]. Unfortunately, we did not analyses plasma anabolic steroids in our experiment. In the present work, the higher IGF-1 levels found in IC males vs. SC males and IC females would enable a higher potential for lean growth in IC males. Indeed, we previously reported higher growth rates and protein depositions in IC males vs. SC males before the second vaccination, with IC females in intermediate positions [18]. Two weeks after the second vaccination, differences between groups were not significant, although N retention and the efficiency of N retention were still numerically higher in IC males. In addition, the plasmatic concentration of IGF-1 of all pigs fed the greater-protein-content diet was also the highest, in agreement with increased N retention as dietary protein increased [18]. In this sense, higher protein synthesis rates in parallel with increased plasmatic IGF-1 levels were reported for IC males fed at a high feed intake, compared to IC males maintained at a lower one [34]. 

Despite possible dissimilarities in dietary protein utilization, no differences in postabsorptive plasma urea concentration were detected between pig types. Nevertheless, plasmatic urea increased progressively as dietary protein intake was augmented, reflecting an enhanced N metabolism as the pigs ingested higher AA amounts [53]. 

Although plasma free AA is a complex pool resulting from inputs from absorbed AA and AA released by tissues (proteolysis and de novo synthesis), and outputs to AA oxidation and metabolism (synthesis of proteins and other molecules) [54], it may provide insights into metabolic mechanisms regarding plasma AA homeostasis and AA utilization by tissues, and therefore, differences in growth potential. Differences in plasma AA concentration between pig types were moderate in the present case, although the trend towards a higher concentration of BCAA and total essential AA in the plasma of IC males can be related to a higher AA availability and potential for lean growth compared with SC males and IC females.

It could be speculated that lower plasma histidine concentrations in SC vs. IC males could be related to the use of histidine in IC as a precursor of other molecules (carnosine, with antioxidant activity, or histamine that influences vasodilatation, blood pressure, mucus secretion, etc. [55]). Proline, one of the major components of collagen [56], was decreased in IC males, which might indicate increased utilization for collagen synthesis in IC males. Muscles from entire males are richer in collagen than those from SC males [57], although no differences in collagen content have been reported between IC and SC male pigs. 

## 5. Conclusions

The postabsorptive plasma parameters of Iberian SC males showed a higher predisposition to carcass fatness and insulin resistance compared to Iberian IC males, which presented trends for increased plasma concentration of branched chain AA and total essential AA in conjunction with higher plasmatic levels of IGF-1. IC females remained in an intermediate situation. The present results support the improved performance and nitrogen retention capacity previously observed in IC male Iberian pigs compared to SC males and IC females, which can be attributed to the increased anabolic capacity related to lean growth in IC males 5 weeks after immunocastration. These findings could be of relevance for Iberian and other indigenous pig breeds for increasing animal welfare, avoiding boar taint, and benefiting from a period of enhanced anabolic capacity in immunocastrated animals. Nevertheless, this metabolic stage might be altered when fattening is prolonged to higher BW (160 kg of higher), which is a common slaughter weight of Iberian pigs. When prolonged fattening is expected, the vaccination protocol should be adapted to include a second revaccination. Further studies are needed to clarify these aspects. 

## Figures and Tables

**Table 1:** Effect of immunocastration and dietary protein level on plasma metabolites and hormone concentrations of male and female Iberian pigs.

	Sex Group ^1^			Dietary Protein ^2^				*p*-Value ^3^	
	IC Males	SC Males	IC Females	LP	MP	HP	SEM	Sex	Dietary Protein
Plasma metabolites									
Glucose, mg/100 mL	159	192	177	189 ^a^	155 ^b^	185 ^a^	11	0.077	<0.05
Triglycerides, mg/100 mL	67.2	66.9	63.5	57.5	73.4	66.7	6.9	0.918	0.278
Ammonia, µM/L	356	338	398	345	368	380	30	0.368	0.708
Total cholesterol, mg/100 mL	121	141	132	132	136	126	5.6	0.063	0.505
HDL-cholesterol, mg/100 mL	62.2	71.0	65.0	69.6	67.1	61.3	2.7	0.066	0.099
LDL-cholesterol, mg/100 mL	41.6 ^b^	49.9 ^a^	46.3 ^ab^	44.9	47.2	45.8	1.9	<0.05	0.685
Creatinine, mg/100 mL	1.50	1.40	1.48	1.45	1.46	1.48	0.03	0.055	0.813
Urea, mg/100 mL	42.2	43.2	43.6	36.8 ^c^	43.3 ^b^	49.0 ^a^	1.2	0.745	<0.001
Plasma hormones									
Insulin, μunits/mL	10.7 ^b^	22.7 ^a^	15.5 ^ab^	15.8	14.2	19.0	3.1	<0.05	0.546
IGF-1, ng/mL	298 ^a^	206 ^b^	237 ^b^	234 ^b^	230 ^b^	277 ^a^	12.8	<0.001	<0.05
Leptin, ng/mL	36.2 ^a^	55.9 ^b^	63.7 ^b^	50.7	56.0	49.1	5.7	<0.01	0.653
Total ghrelin, pg/mL	329	267	263	283	298	277	21.3	0.057	0.801
Insulin sensitivity indexes ^4^									
HOMA-IR	5.3 ^b^	15.3 ^a^	9.1 ^ab^	9.6	7.3	12.8	2.7	<0.05	0.350
HOMA-%B	34.1	42.0	33.7	32.6	41.7	35.6	6.8	0.609	0.607
QUICKI	0.308 ^a^	0.278 ^b^	0.289 ^ab^	0.287	0.299	0.288	0.007	<0.01	0.340

^1^ IC males = immunocastrated males, SC males = surgically castrated males, IC females = immunocastrated females; *n* = 54 pigs (9 treatments × 6 pigs per treatment). ^2^ LP = low CP diet (119 g CP/kg DM); MP = medium CP diet (137 g CP/kg DM); HP = high CP diet (153 g CP/kg DM). ^3^ Interaction sex group × dietary crude protein (CP) was never significant. ^4^ HOMA-IR, Homeostasis Model Assessment for estimating insulin resistance; HOMA-%B, Homeostasis Model Assessment for estimating β-cell function; QUICKI, Quantitative Insulin Sensitivity Check Index. a–c Values within a row with different superscripts differ significantly at *p* < 0.05.

**Table 2:** Effects of immunocastration and dietary protein level on plasma free amino acid (AA) concentrations (µmol/L) of male and female Iberian pigs.

	Sex Group ^1^			Dietary Protein ^2^				*p*-Value ^3^	
	IC Males	SC Males	IC Females	LP	MP	HP	SEM	Sex	Dietary Protein
Essential AA									
Arginine	102	99	107	106	106	96	6	0.634	0.441
Histidine	37 ^a^	28 ^b^	33 ^ab^	34	33	31	2	<0.01	0.498
Isoleucine	228 ^a^	217 ^a^	192 ^b^	210	210	217	7	<0.01	0.657
Leucine	249	259	245	254	251	248	8	0.406	0.887
Lysine	161	172	163	172	176	147	9	0.618	0.051
Methionine	25	26	22	26	26	21	2	0.335	0.105
Phenylalanine	50	49	51	51	50	49	2	0.699	0.427
Threonine	80	84	81	84	82	80	6	0.846	0.838
Tryptophan	11	11	12	13	11	11	1	0.954	0.453
Valine	564 ^a^	501 ^b^	460 ^b^	508	507	510	18	<0.001	0.992
Branched-chain AA	1041 ^a^	977 ^ab^	897 ^b^	971	968	976	30	<0.01	0.984
Σ essential AA	1507	1447	1364	1457	1452	1408	47	0.096	0.705
Non-essential AA									
Alanine	335 ^b^	345 ^b^	412 ^a^	357	390	344	22	<0.05	0.300
Anserine	112	103	113	108	118	102	6	0.446	0.191
Aspartic acid	14	15	14	16	14	14	1	0.713	0.348
Glutamine	205	204	221	214	221	195	10	0.402	0.155
Glutamic acid	441	495	502	518	470	450	28	0.225	0.207
Glycine	446	463	527	470 ^b^	539 ^a^	426 ^b^	27	0.093	<0.05
Hydroxiproline	23	27	25	24	26	25	2	0.386	0.827
Ornithine	38	42	42	42	43	36	3	0.410	0.131
Proline	210 ^b^	296 ^a^	307 ^a^	270	294	249	25	<0.05	0.463
Phosphoserine	11 ^c^	16 ^b^	25 ^a^	18	17	17	2	<0.001	0.939
Serine	107	104	119	107	115	108	5	0.099	0.521
Taurine	131 ^b^	174 ^a^	168 ^a^	152	164	157	7	<0.001	0.446
Tyrosine	67	72	72	75	71	65	3	0.409	0.055
Σ non-essential AA	2141 ^b^	2355 ^ab^	2547 ^a^	2372	2482	2189	91	<0.01	0.072
Proteinogenic AA	3232	3340	3431	3379	3460	3163	106	0.398	0.122
Non-proteinogenic AA	417 ^b^	461 ^ab^	480 ^a^	449	474	434	18	<0.05	0.281
Σ total AA	3648	3801	3911	3829	3934	3597	121	0.287	0.126

^1^ IC males = immunocastrated males, SC males = surgically castrated males, IC females = immunocastrated females; *n* = 54 pigs (9 treatments × 6 pigs per treatment). ^2^ LP = low CP diet (119 g CP/kg DM); MP = medium-CP diet (137 g CP/kg DM); HP = high-CP diet (153 g CP/kg DM). ^3^ Interaction sex group × dietary crude protein (CP) was never significant. a–c Values within a row with different superscripts differ significantly at *p* < 0.05.

**Table 3:** Effect of immunocastration and dietary protein level on total lipid content and fatty acid (FA) composition of backfat of male and female Iberian pigs (g FA/100 g of identified FA methyl esters).

	Sex Group ^1^			Dietary Protein ^2^				*p*-Value ^3^	
	IC Males	SC Males	IC Females	LP	MP	HP	SEM	Sex	Dietary Protein
Lipids, g/100 g	92.0 ^b^	94.1 ^a^	93.3 ^a^	93.1	93.2	93.1	3.3	<0.001	0.967
C10:0	0.011	0.012	0.013	0.011	0.012	0.013	0.001	0.249	0.128
C12:0	0.041	0.045	0.046	0.042	0.043	0.047	0.002	0.142	0.123
C14:0	1.21 ^b^	1.31 ^a^	1.25 ^ab^	1.25	1.23	1.29	0.03	<0.05	0.270
C16:0	26.4	26.7	26.7	26.7	26.5	26.7	0.27	0.593	0.772
C16:1	1.75	1.72	1.72	1.73	1.63	1.82	0.07	0.930	0.136
C17:0	0.449	0.435	0.394	0.427	0.425	0.425	0.023	0.204	0.996
C18:0	16.1	16.0	16.6	16.5	16.5	15.7	0.39	0.558	0.256
C18:1 n-9	41.2	41.6	40.9	41.1	41.3	41.5	0.40	0.441	0.825
C18:1 n-7	2.45 ^a^	2.42 ^a^	2.15 ^b^	2.33	2.31	2.38	0.06	<0.01	0.651
C18:2 n-6	7.14 ^a^	6.43 ^b^	6.90 ^a^	6.77	6.83	6.87	0.14	<0.01	0.878
C18:3 n-3	0.505	0.567	0.507	0.474 ^b^	0.536 ^ab^	0.569 ^a^	0.027	0.164	<0.05
C20:0	0.250	0.248	0.272	0.267 ^a^	0.267 ^a^	0.236 ^b^	0.008	0.085	<0.05
C20:1 n-9	1.25	1.30	1.32	1.30	1.29	1.28	0.05	0.483	0.942
C20:2 n-6	0.410 ^ab^	0.383 ^b^	0.425 ^a^	0.398	0.413	0.407	0.012	<0.05	0.669
C20:3 n-6	0.041 ^b^	0.042 ^b^	0.054 ^a^	0.045	0.044	0.047	0.002	<0.001	0.451
C20:4 n-6	0.087 ^ab^	0.073 ^b^	0.098 ^a^	0.079	0.089	0.091	0.006	<0.05	0.322
C20:5 n-3	0.006	0.007	0.006	0.006	0.006	0.007	0.0005	0.145	0.328
C22:0	0.016 ^b^	0.017 ^b^	0.025 ^a^	0.021	0.019	0.018	0.002	<0.01	0.432
C22:4 n-6	0.026 ^b^	0.033 ^a^	0.036 ^a^	0.032	0.032	0.031	0.002	<0.01	0.974
C22:5 n-3	0.023	0.026	0.022	0.021 ^b^	0.023 ^b^	0.027 ^a^	0.001	0.161	<0.05
Total SFA ^4^	45.0	45.2	45.7	45.6	45.4	44.9	0.6	0.673	0.673
Total MUFA	46.7	47.1	46.2	46.5	46.5	47.0	0.5	0.636	0.733
Total PUFA	8.34 ^a^	7.66 ^b^	8.17 ^a^	7.93	8.09	8.16	0.16	<0.01	0.575
Total n-6	7.73 ^a^	6.98 ^b^	7.54 ^a^	7.35	7.43	7.46	0.15	<0.01	0.838
Total n-3	0.62	0.68	0.63	0.58 ^b^	0.65 ^ab^	0.69 ^a^	0.03	0.264	<0.05
n-6:n-3	13.3 ^a^	10.8 ^b^	12.1 ^ab^	13.4 ^a^	11.6 ^b^	11.1 ^b^	0.6	<0.01	<0.05
PUFA/SFA	0.188	0.170	0.180	0.177	0.179	0.183	0.006	0.095	0.759
MUFA/SFA	1.05	1.05	1.02	1.03	1.03	1.05	0.03	0.546	0.795

^1^ IC males = immunocastrated males, SC males = surgically castrated males, IC females = immunocastrated females; *n* = 54 pigs (9 treatments × 6 pigs per treatment). ^2^ LP = low CP diet (119 g CP/kg DM); MP = medium-CP diet (137 g CP/kg DM); HP = high-CP diet (153 g CP/kg DM). ^3^ Interaction sex group × dietary crude protein (CP) was never significant. ^4^ SFA: saturated fatty acids; MUFA: monounsaturated fatty acids; PUFA: polyunsaturated fatty acids. a,b Values within a row with different superscripts differ significantly at *p* < 0.05.

## Data Availability

The datasets that support the findings of this study are available from the corresponding author upon request.

## Supplementary Materials

The following are available online at https://www.mdpi.com/article/10.3390/ani13162650/s1, Table S1: Ingredients and nutrient composition of experimental diets. Reference [58] are cited in the supplementary materials.
