# Supplementary material for: Metabolic Profile of Growing Immune- and Surgically Castrated Iberian Pigs Fed Diets of Different Amino Acid Concentration
Source: Animals (Basel). 2023 Aug 17;13(16):2650. doi: 10.3390/ani13162650 (PMC10451894; doi:10.3390/ani13162650)
Supplement: Supplementary file 1 [file animals-13-02650-s001.zip › animals-2493716-supplementary.docx]

**Supplementary Table S1.** Ingredients and nutrient composition of experimental diets

|  | **Experimental diets^1^** | | | |
| --- | --- | --- | --- | --- |
|  | | **LP** | **MP** | **HP** |
| Ingredients, g/kg as fed | | | |  |
| Barley grain | | 700 | 700 | 700 |
| Corn | | 242 | 199 | 156 |
| Soybean meal, 47% CP | | 27 | 70 | 113 |
| Monocalcium phosphate | | 10.2 | 9.3 | 9.0 |
| Calcium carbonate | | 6.1 | 6.2 | 6.3 |
| NaCl | | 3.0 | 3.0 | 3.0 |
| Vitamin/mineral pre-mix^2^ | | 3.0 | 3.0 | 3.0 |
| L-lysine, 50% | | 6.2 | 6.2 | 6.3 |
| L-threonine, 50% | | 2.2 | 2.4 | 2.6 |
| Methionine hydroxy analoge, 75% | | 0.42 | 0.6 | 0.8 |
| L-tryptophan, 19.6% | | 0.30 | 0.07 | 0.0 |
| Analysed nutrient composition, g/kg DM | | | |  |
| Crude protein | | 119 | 137 | 153 |
| Lysine | | 7.8 | 9.1 | 10.4 |
| Methionine | | 2.2 | 2.6 | 3.0 |
| Methionine + Cysteine | | 4.6 | 5.2 | 5.9 |
| Threonine | | 5.1 | 6.0 | 6.9 |
| Tryptophan^3^ | | 1.3 | 1.6 | 1.8 |
| Lipids | | 27 | 28 | 27 |
| Total ash | | 53 | 58 | 62 |
| Gross energy (MJ/kg DM) | | 18.03 | 17.96 | 17.99 |

^1^LP = low crude protein (CP) diet (119 g CP/kg DM); MP = medium CP diet (137 g CP/kg DM); HP = high CP diet (153 g CP/kg DM).

^2^ Provided (per kg of diet): 2000 UI retinol as retinyl acetate, 800 UI cholecalciferol, 40 UI dl-α-tocopheryl acetate, 1.5 mg menadione, 2 mg thiamine, 3 mg riboflavin, 50 μg cyanocobalamin, 15 μg folic acid, 22.5 mg nicotinic acid, 15 mg d-pantothenic acid, 60 mg MnO, 80 mg FeCO_3_, 80 mg ZnO, 750 μg KI, 10 mg CuSO_4_.5H_2_O, 50 μg Na_2_SeO_3_, 250 mg sepiolite, 1.5 mg butylhydroxyanisole(BHA) and 7.5 mg butylhydroxytoluene (BHT).

^3^ Calculated [58].
